# Utilization of Biased G Protein-Coupled Receptor Signaling towards Development of Safer and Personalized Therapeutics

**DOI:** 10.3390/molecules24112052

**Published:** 2019-05-29

**Authors:** Metehan Ilter, Samman Mansoor, Ozge Sensoy

**Affiliations:** 1 Department of Biomedical Engineering, The School of Engineering and Natural Sciences, Istanbul Medipol University, Istanbul 34810, Turkey; milter@st.medipol.edu.tr; 2 Department of Biomedical Engineering and Bioinformatics, The Graduate School of Engineering and Natural Sciences, Istanbul Medipol University, Istanbul 34810, Turkey; smansoor@st.medipol.edu.tr; 3 Department of Computer Engineering, The School of Engineering and Natural Sciences, Istanbul Medipol University, Istanbul 34810, Turkey

**Keywords:** GPCR, biased signaling, allostery, personalized medicine, functional selectivity, oligomerization, single nucleotide polymorphism, arresin, G protein

## Abstract

G protein-coupled receptors (GPCRs) are involved in a wide variety of physiological processes. Therefore, approximately 40% of currently prescribed drugs have targeted this receptor family. Discovery of β-arrestin mediated signaling and also separability of G protein and β-arrestin signaling pathways have switched the research focus in the GPCR field towards development of biased ligands, which provide engagement of the receptor with a certain effector, thus enriching a specific signaling pathway. In this review, we summarize possible factors that impact signaling profiles of GPCRs such as oligomerization, drug treatment, disease conditions, genetic background, etc. along with relevant molecules that can be used to modulate signaling properties of GPCRs such as allosteric or bitopic ligands, ions, aptamers and pepducins. Moreover, we also discuss the importance of inclusion of pharmacogenomics and molecular dynamics simulations to achieve a holistic understanding of the relation between genetic background and structure and function of GPCRs and GPCR-related proteins. Consequently, specific downstream signaling pathways can be enriched while those that bring unwanted side effects can be prevented on a patient-specific basis. This will improve studies that centered on development of safer and personalized therapeutics, thus alleviating the burden on economy and public health.

## 1. Introduction

G protein-coupled receptors (GPCRs) constitute large protein families having more than 800 members [1,2]. They mediate various crucial signaling pathways which makes them one of the most targeted molecules in the drug market: 40% of currently prescribed drugs target GPCRs [1,3]. However, this is not a trivial task since GPCRs share a common 3D structure with high sequence similarity in particular for the orthosteric ligand binding site among receptor subtypes.

**Figure 1:**
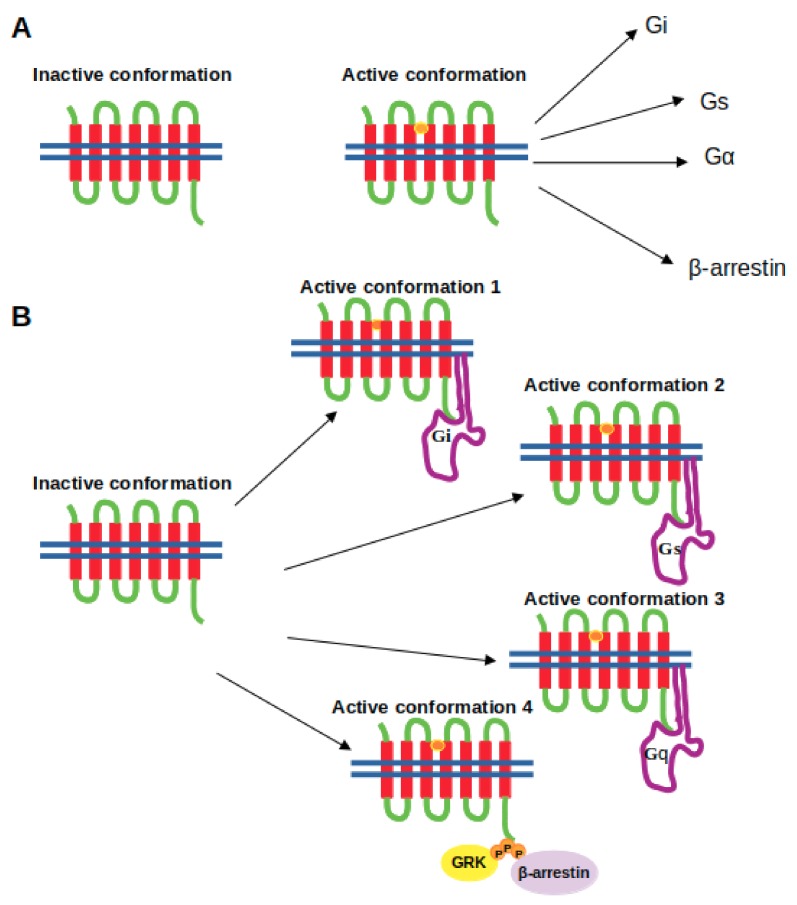
Depiction of the (**A**) the two-state model and (**B**) the “new-model” proposed for GPCR activation. The ligand is shown in the orange circle, whereas the boundary of the cell membrane is shown by blue lines.

Discovery of β-arrestin-mediated signaling, which is independent of G protein, and separability of these two signaling pathways have opened up a new platform for development of safer and selective drugs and switched the focus in the GPCR field to the discovery of effective biased ligands, which can enrich a certain signaling pathway while preventing the others that cause unwanted side effects [4,5,6,7,8]. This discovery has also changed the view of “two-state” or “binary-switch” model [9,10,11,12,13], which was proposed for activation of GPCRs. In the “old-model”, it is proposed that GPCRs can only adopt one active conformational state to which various types of signalling effectors can bind. On the other hand, in the new view, GPCRs can be modelled as allosteric microprocessors which generate a vast number of conformations depending on the pharmacologic properties of ligands (see Figure 1). For instance, full agonists lead to a large shift in the conformational equilibrium of both transmembrane (TM) 6 & 7, whereas partial agonists have a smaller influence on TM6. On the other hand, β-arrestin biased ligands strongly impact conformational equilibrium of Helix 8 [14].

Studies that focus on development of biased ligands have started with targeting the orthosteric ligand binding sites [15,16]. As mentioned above, conservation of this primary ligand binding pocket has necessitated the exploration of alternative regions on the receptor such as allosteric regions which are relatively less conserved and involved in activation mechanisms of the receptor [17]. Later on, more sophisticated ligands such as bitopic ligands have been developed that can simultaneously bind to the orthosteric and allosteric site of the receptor, thus further increasing the selectivity [18,19]. Moreover, the progress in computational power and development of enhanced sampling algorithms have provided discovery of additional regions like metastable sites on the receptor which can be targeted together with the orthosteric ligand binding site [20]. However, studies have shown that single nucleotide polymorphisms have been seen in above-mentioned functional regions of GPCRs and they are associated with variations in drug responses [21,22,23]. Therefore, inclusion of pharmacogenomics as a routine in drug discovery studies will improve efficacy of drugs and also build up a fruitful platform for development of successful personalized medicine.

Besides the transmembrane region, intracellular regions of GPCRs have been also targeted by means of special class of molecules such as pepducins and RNA aptamers due to lower sequence similarity seen at that region than the transmembrane [24,25,26,27,28]. Moreover, as an example of intracellular biasing, GPCR-related proteins such as G proteins, β-arestins as well as G protein-coupled receptor kinases (GRKs) can be targeted as well which requires information regarding the activation mechanism of these proteins as well as interaction interfaces formed between the effector and GPCR. Last but not least, oligomerization also impacts signaling properties of GPCRs under both normal and disease conditions [29,30,31]. Interestingly, in disease conditions, drug treatment also impacts the identity of GPCR oligomers [32,33,34].

In this review, we discuss the role of biased signaling in development of safer therapeutics and summarize the current state of strategies that are used to modulate signaling properties of GPCRs. We also point out the importance of inclusion of pharmacogenomics and molecular dynamics simulations in drug discovery studies . Therefore, this review should provide a cutting edge perspective for researchers whose studies focus on development of safer personalized therapeutics that target GPCRs.

## 2. Biased Signaling

**Figure 2:**
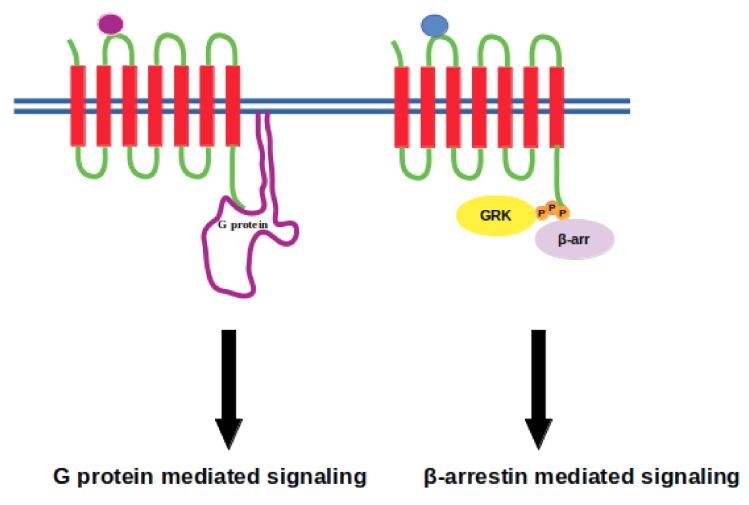
Purple and blue beads represent biased ligands where they initiate either G protein or β-arrestin mediated signaling, respectively. G protein-coupled receptor kinase is denoted by GRK and represented with yellow circle that phosphorylates the intracellular domain of the receptor, whilst β-arrestin is represented with a purple circle.

GPCR signaling was thought to be mediated solely by G proteins until the discovery of β-arrestin mediated signaling, which is independent of G proteins, in addition to its well-established role in termination of signaling cascade [35,36] (see Figure 2). Briefly, ligand binding to GPCR causes a set of conformational changes in the receptor which can be recognized by the heterotrimeric G protein. G protein is then recruited from cytosol to the membrane, binds to the receptor and is activated. Upon activation, it dissociates from the receptor, which is followed by phosphorylation of the receptor via GPCR kinases. Consequently, active-phosphorylated receptor is recognized by another cytosolic protein, namely β-arrestin. It binds to the cytoplasmic site of the receptor and occupies that volume, which is otherwise occupied by G protein. As a result, G protein cannot bind to the receptor and the signal is terminated. Here, the requirement of receptor phosphorylation at a precise site is important as any problem associated with this step prevents Arrestin binding to receptor as in the case of retinitis pigmentosa [37]. Alternatively, extremely high concentration of the extracellular ligand might cause Arrestin to interact more strongly with the receptor, which leads to downregulation as in the case of heart failure [38]. These breakthroughs and the fact of separability of G protein and β-arrestin-mediated pathways have switched the research focus in the GPCR-field towards development of biased ligands, which can initiate a specific downstream signaling pathway, thus paving the way for development of therapeutics with fewer side effects [39,40,41].

From a structural perspective, activation of a certain signaling pathway can be explained by stabilization of a specific conformational state at intracellular region of GPCR to which only G protein or Arrestin can couple. The first evidence for this conformational discrepancy came from an ^19F^NMR study where cytoplasmic ends of transmembrane (TM) 6 and 7 have been shown to adopt two major conformational states depending on the type of the ligand. In particular, G protein biased ligands shift the equilibrium towards the active state of TM6, whereas β-arrestin-biased ligands dominantly affect the conformational state of TM7 [14]. In another recent study, the authors have shown that this conformational difference is even present among ligands that can activate different subtypes of G proteins [42]. From these findings, it is indisputable that GPCRs can adopt more than one active conformational state, which is in contrast to “single active state” theory [43,44,45]. Moreover, each of these conformations provides specific coupling of the receptor with a specific effector, thus initiating a particular signaling pathway [43,45,46,47,48,49].

Due to a strong link between functional selectivity and development of safer therapeutics, biased signaling pathways have been thoroughly studied for some specific GPCRs such as opioid, dopamine (D_2_R), β-adrenergic and angiotensin receptors. Opioid receptors (ORs) have been widely used as targets for analgesics [16,50]; however, OR-targeted drugs cause some side effects such as respiratory, depression, and constipation. Studies have reported that analgesic impact of μ-OR is governed by Gi-mediated signaling, whereas β-arrestin-mediated signaling causes these unwanted outcomes [5,16,51]. With this pharmacological knowledge, drug discovery studies, which have focused on development of G protein biased μ-OR ligands, have been expedited. For instance, TRV130, which acts as a G protein biased μ-OR ligand, has passed phase II trials and has been transferred to phase III [16,52]. Moreover, computational studies have been also conducted to provide more insight into key atomistic interactions formed between the biased ligand and the receptor. In a recent study, the operating mode of activation switch, which is composed of W^6.48^ and Y^7.43^ residues (Ballesteros/Weinstein numbering), and is responsible for down and upregulation of β-arrestin-mediated signaling [16,53], respectively, has been revealed [16]. According to that, W^6.48^ is found to be stabilized by interacting with Y^7.43^ in the presence of a G protein-biased ligand, TRV130. In addition, further stabilization, which is mediated by hydrophobic interactions, is also observed for W^7.35^. Such atomistic-level data can guide design studies of novel biased ligands in a more systematic and precise way.

Apart from ORs, D_2_R has been also studied to delineate structural determinants that provide specific coupling of the receptor to either G protein or β-arrestin. It has been shown that, on animal models of schizophrenia, noncanonical modes of D_2_R signaling via β-arrestin has a therapeutic advantage over G protein-mediated signaling in terms of antipsychotic efficacy and also they are protective against motoric side effects [54,55,56]. McCorvy et al. have elucidated that specific amino acids which are located at EL2 and TM5 region of the receptor mediate β-arrestin- and Gi/o-mediated signaling, respectively. More importantly, it has been also shown that the bias is conserved among other aminergic GPCRs which maintain similar residues at the EL2 and TM5 regions [57].

Additional structurally important regions, namely EL2 of serotonin (5-hydroxytryptamine,5-HT) receptor, have been shown to participate in biased signaling. Specifically, the prototypical hallucinogen Lysergic acid diethylamide (LSD) interacts with EL2 of 5-HT_2B_R, which modulates β-arrestin2 recruitment. Drugs, which target these receptors, are used for treatment of migraine, headaches, schizophrenia, depression, anxiety and obesity [58,59,60]. Another example can be given for β2-adrenergic receptor, which plays a crucial role in normal functioning of the heart. It has been shown that β-arrestin-mediated signaling has some advantages over Gαs-mediated signaling such as cardioprotective effects. Therefore, biased ligands which can specifically trigger β-arrestin-mediated pathway, such as carvedilol, can be used effectively in treatment of heart failure [39,61]. In addition to carvedilol, TRV120027, which acts as a β-arrestin biased ligand at the angiotensin II type 1 receptor (AT_1_R), has been shown to elicit beneficial cardiorenal actions such as increased cardiomyocyte survival rate [62,63]. Moreover, it has been also showed that Sar1, Ile4, Ile8-angiotensin (SII), which acts on AT1aR, does not activate Gαs-dependent signaling pathway, yet it recruits specifically β-arrestin and activates ERK as well as other biochemical effectors which are involved in β-arrestin-dependent behavior, suggesting that SII can be used as a reference ligand to decipher β-arrestin biased agonism in other GPCRs [39,64]. Last but not least, it is also important to emphasize that results of biased signaling studies might be different depending on the type of the organism used. For instance, HU910 and HU308, which are CB_2_ selective ligands, displayed well-balanced agonist profiles in humans, whereas they displayed biased agonism in favor of G protein in mouse CB_2_ [65,66].

In the following section, allosteric modulators of biased GPCR signaling will be summarized with an emphasis on bitopic ligands and the oligomerization phenomenon.

## 3. Allosteric Modulators of Biased GPCR Signalling

Besides orthosteric ligands, allosteric modulators are also capable of inducing conformational changes in GPCRs, which can govern the structure of both orthosteric ligand binding and effector coupling site [67]. Therefore, they impact GPCR signaling by modulating effects which are exerted by orthosteric ligands. Here, it is important to emphasize that effects of allosteric modulators on ligand affinity and efficacy might not be always unidirectional, which depends on cooperativity between orthosteric and allosteric modulators [68]. That is to say, the modulator can decrease the efficacy while increasing the affinity or vice versa. Recent studies have shown that usage of allosteric modulators, rather than traditional orthosteric ligands, results in an increase in both safety and differential selectivity [17]. Presumably, this is due to the fact that allosteric sites exhibit vast divergence in their primary sequence among species whilst orthosteric binding sites are more conserved. However, allosteric modulators with restricted cooperativity may cause a decrease in efficacy profiles [68], which leads to administration of higher dosage of the drug and so toxicity in the cell, which is known as the “ceiling effect” [19]. This phenomenon is also crucial for discriminating subtypes of a given receptor [25,69,70]. Consequently, usage of orthosteric ligands are hindered due to insufficient subtype selectivity as shown for mAChR, which is targeted to combat with Alzheimer’s disease, schizophrenia, and drug addiction [25,71,72]. In addition, allosteric modulators not only fine-tune receptor signaling but also attenuate risk of overdosing, which is relevant to saturation [25,73,74,75]. For instance, allosteric regulation of cannabinoid receptor 1 (CB_1_) enables more precise control of downstream pathways [76]. Moreover, insurmountable character of allosteric ligands is also important, which is known as ability of allosteric ligand to decrease the potency and/or efficacy of the endogenous agonist even under high concentration [77]. Le et al. have illustrated that olmesartan, as opposed to most antagonists, displays a high degree of insurmountability for angiotensin II type 1 (AT_1_) receptor, thus affecting the maximal response on the concentration–response curve [78]. Consequently, it can be thought that allosteric modulators can be used to mask detrimental pathways while augmenting beneficial ones. However, it must also be kept in mind that allosteric regulation of GPCRs might bring undesirable side effects as well [67] unless pharmacology of circulating metabolites, which are formed by breakdown of allosteric ligands, is considered. In addition, allosteric ligands might be highly polyaromatic or lipophilic, which makes it difficult to work with them because of low solubility [73].

Allosteric modulators can be grouped into three classes, namely, positive, negative, and neutral which are denoted by PAM, NAM and NAL, respectively. PAMs introduce bias into signaling pathway which cannot be induced by natural ligands; for instance, phosphotidylglycerol increases agonist binding affinity and triggers receptor activation for β2-adrenergic receptor (β2R), whereas phosphatidylethanolamine makes antagonist binding favorable for β2R, which results in stabilization of the inactive state of the receptor [79]. In another study, PAMs were utilized to stabilize binding of antagonists and to induce selectivity among different subtypes of muscarinic acetylcholine receptors [80]. In particular, LY2033298 and thiochrome have been shown to exhibit higher selectivity for M_4_mAChR than other subtypes [25,75,81,82]. NAMs, on the other hand, preclude activation of receptor by binding to allosteric site. It is also crucial to stress that NAMs lower the binding efficacy and affinity of orthosteric agonists via negative cooperativity [83].

As opposed to PAMs and NAMs, NALs bind to the allosteric sites and they change neither binding affinity nor efficacy of the orthosteric ligands [73]. For instance, 5-methyl-6-(phenylethynyl)-pyridine, which is close analog of 2-methyl-6-(phenylethynyl)-pyridine, acts as an allosteric ligand on metabotropic glutamate receptor subtype 5, and it displays only partial inhibition or no functional effects on the mGlu5 response [84].

In addition to chemical modulators cations also act as allosteric modulators. In a recent study, it has been shown that high concentration of divalent cations, namely Mg^2+^, Ca^2+^, and Mn^2+^, triggers A_2A_ adenosine activation by bringing TM5 and TM6 together at the extracellular region of the receptor while high concentration of Na^+^, which is a modulator of the activity of a large number of GPCRs [85], causes receptor inactivation [27]. Another cation, namely Zn^2+^, acts as a negative allosteric inhibitor on 5-HT_7_ receptors [86]. Moreover, surrounding lipids also mediate function of GPCRs and impact of lipids is modulated by the type of the receptor. For instance, cholesterol stimulates agonist binding to oxytocin and serotonin receptors while it triggers dimerization of NTS1 receptor [79,87,88,89]. In another study, lipid head groups have been shown to modulate also activity of β2R and chemokine receptor [79,90].

Apart from transmembrane region and surrounding medium, the intracellular region of GPCRs, to which effectors couple, have also been targeted for more than a decade by means of pepducins and RNA aptamers. As to pepducins, which are cell-penetrating peptides, in particular, KRX-725 targets Spingosine-1-phosphate receptor subtype 3 (S1P3) to induce fibroblast proliferation and vasorelaxation by mimicking the effect of sphingosine-1-phosphate [91]. In addition, a study conducted by Carr et al. has indicated that β-arrestin-biased pepducin that targets β_2_AR can be used to prevent congestive heart failure [92]. Despite their receptor selectivity and capability of initiating a specific signaling pathway, the precise mechanism of action of this class of ligands remains elusive, thus making design of effective molecules difficult. Moreover, degree of hydrophobic character of these molecules is also crucial since they have to cross the cell membrane to act. RNA aptamers, on the other hand, are made up of nucleotides, rather than amino acids, and they display high affinity and specificity. It has been shown that targeted disruption of βarrestin2-mediated signaling pathways by aptamer chimeras can be used to inhibit leukemic cell growth [93]. Last but not least, voltage can also be given as an example to allosteric modulators. In a molecular dynamics study of muscarinic receptor, it has been shown that M_3_ and M_1_-R display subtype specific crosstalk between their orthosteric and allosteric sites upon stimulation by voltage [94].

Advantages and superiority of allosteric ligands over orthosteric ones are discussed in detail above. The importance of cooperativity between allosteric and orthosteric ligand binding pockets, which is required for effective allosteric ligand activity, is also mentioned. In light of these findings, researchers have been developing specific class of ligands, namely bitopic ligands, that can occupy both orthosteric and allosteric ligand binding sites of the receptor, which will be discussed in detail below.

### 3.1. Bitopic Ligands

**Figure 3:**
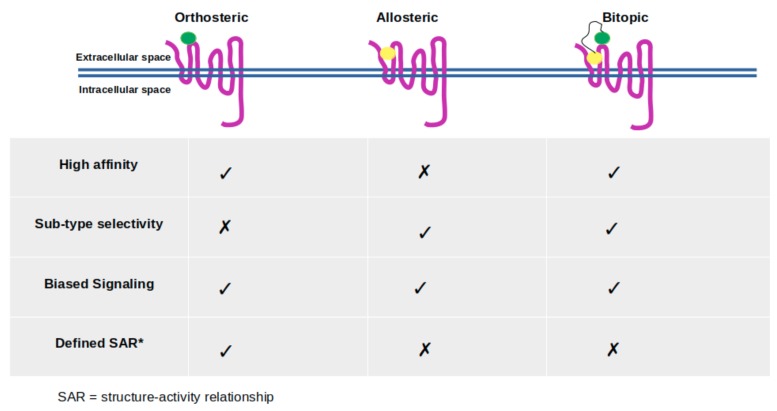
The orthosteric ligand, which is shown with green beads, targets orthosteric binding sites of the receptor, whereas the allosteric ligand, which is shown with yellow beads, targets a topologically different site named allosteric site. Bitopic ligand consists of two ligands that target both orthosteric and allosteric sites. The relevant key signaling properties for three types of targeting are indicated—inspired by Kruse et al. [72].

Bitopic (dualsteric) ligands consist of two pharmacophores, which are specifically designed for simultaneous targeting of orthosteric and allosteric ligand binding sites located on the same receptor, and they are linked by a chemical group [20,95] (see Figure 3). As mentioned above, orthosteric binding sites are highly conserved among subtypes of the same GPCR, thus targeting this site may cause selectivity problems [20,67,96]. Therefore, bitopic ligands are more likely to cope with this problem as they simultaneously target both sites [20,25]. In addition, selectivity, bitopic ligands exhibit high affinity and trigger biased signaling as well [20,97,98].

Improvements in computational power and development of sophisticated sampling algorithms have enabled the scientific community to achieve time scales (currently up to microseconds) which are accessible in experiments. Consequently, novel conformational states such as metastable binding site and/or interaction sites that have been revealed by crystallographic and spectroscopic studies [99,100], and concurrently, have been consistently observed in atomistic molecular dynamics simulations as well [20,101,102,103,104,105,106].

Sphingosine-1 phosphate receptors (S1PRs) mediate immune cell regulation and development and vasculogenesis. Non-selective S1PR modulator fingolimod is used to treat multiple sclerosis; however, it cannot differentiate between different subtypes of the receptor [20,107]. On the other hand, bitopic SPM-354, which acts as an antagonist, can selectively target S1PR subtype 3, and it displays enhanced potency and in vivo efficacy such that it rescues cells from complete heart block [20,107,108,109]. Apart from this study, SB269652, which is another bitopic ligand, is capable of discriminating dopamine D_2_ receptor monomers and homodimers [110,111,112]. Moreover, other studies have focused on biased signaling property that is induced by bitopic ligands. It has been reported that trans-8-OH-PBZI and PD128097 induce biased signaling by means of allosteric interactions which are formed within the secondary binding pocket of D_3_R. Consequently, this increases functional selectivity (trans-8-OH-PBZI) and mediates slow response termination properties and tolerance (PD128097) [113,114,115,116,117,118]. In addition, by using 2-amino-3-benzolythiophene allosteric modulator as a reference, bitopic VCP746 has been designed to target adenosine A1 receptor, which is a major therapeutic target for cardio protection [119]. It displays biased agonism relative to prototypical A_1A_R ligands and protects against ischemic insult in native A_1A_R-expressing cardiomyoblasts and cardiomyocytes, while maintaining physiological rat atrial heart rate [119]. In another interesting study, it has been shown that the signaling state of the receptor is modulated by different binding poses of the pharmacophore groups. As an example, the bitopic ligand iper-6-naph, which is comprised of 6-naph and iperoxo groups, can be given. It targets M_2_R and binds in two different modes [120,121]. In one of them, 6-naph group binds to the allosteric site while the iperoxo group binds to the orthosteric site of the receptor, thus leading to signaling through G protein mediated pathways. However, in the second binding mode, 6-naph binds to the orthosteric site, whereas the iperoxo group binds to the allosteric site, thus preventing activation of the receptor. These two binding modes as well as the active and inactive receptor states are co-found within the same ensemble, suggesting that this might affect the efficacy and the therapeutic dosage of the ligand administrated [95,121,122,123].

**Figure 4:**
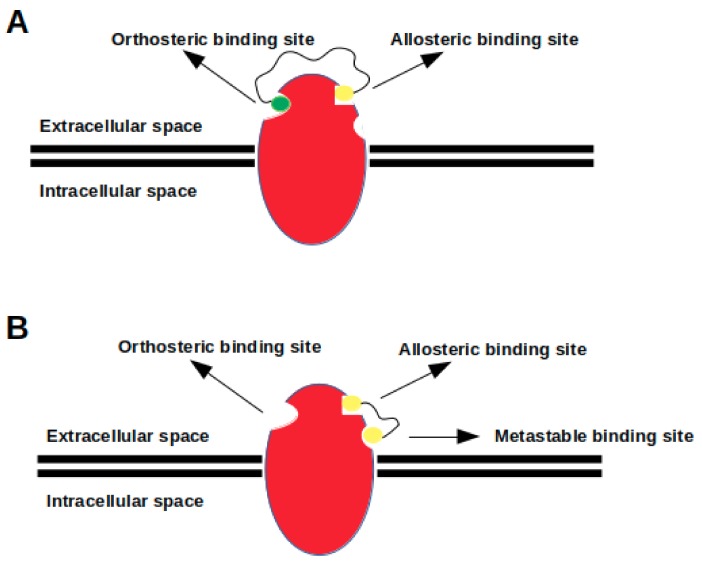
(**A**) a heterobivalent ligand, which is composed of two distinct pharmacophore groups (yellow and green beads) connected by a linker, targets both orthosteric and allosteric binding site simultaneously; (**B**) a homobivalent ligand, which consists of two identical pharmacophore groups (two yellow beads) linked by a proper linker, targets both allosteric and metastable binding sites simultaneously.

In spite of being widely used, development of bitopic ligands poses a challenge, since it is not trivial to determine relevant allosteric binding sites on the target molecule [20,72]. In addition, relation between structure and function of bitopic ligands has not been established yet (See Figure 4), which makes it hard to predict physiological outcome elicited by these ligands. For instance, a bitopic ligand that targets M_1_ mAChR is designed to act as both a PAM and a full agonist; however, studies showed that it acted as a partial agonist [20,124]. Furthermore, bitopic ligands which are composed of pharmacophore groups having opposite pharmacological properties, such as NAM-agonist hybrid, might alleviate one another’s affinity since each pharmacophore might likely to stabilize a set of distinct receptor conformations [20,125]. Lastly, bitopic ligands might also impact oligomerization dynamics of GPCRs by forming stable preexisting dimers for prolonging their lifetime [112,126,127].

In the following section, computational and experimental studies that focused on metastable sites will be reviewed.

#### Metastable Binding Sites

Compared to highly conserved orthosteric sites, metastable binding sites are poorly conserved. Therefore, research focus has shifted towards development of bivalent ligands, which are made from two pharmacophore groups, that can simultaneously bind to orthosteric and metastable sites on the receptor [20]. Consequently, high affinity and selectivity are achieved as also seen for bitopic ligands. However, these ligands are made up of two identical groups, so they are also also known as homobivalent ligands.

Binding kinetic experiments have shown that metastable binding sites act as selectivity filters and this property is explored in ligand entry pathways of serotonin and dopamine receptors [20,128] where metastable sites have been shown to differ among different subtypes of the same receptor. Specifically, an inverse agonist tiotropium binds to a metastable site of M_3_ mAChR by interacting with Leu225^ECL2^ and Phe211^ECL2^, whereas it interacts with Phe181 and Tyr177 when it binds to M_2_ mAChR [20,123]. In another study, the metastable site of Taste receptor type 2 member 46 (TAS2R46) bitter taste receptor for agonist strychinene was shown to be located in the vicinity of ECL1 [20,129]. In addition, metastable binding site of P2Y_12_ receptor has been shown to mediate conformational rearrangements which are necessary for binding of antagonist ticagrelor to the orthosteric site of the receptor [20,103]. Therefore, it can be concluded that conformational rearrangements induced by homobivalent ligands might help engagement of ligands with the orthosteric binding pocket [20,128,130]. In a recent computational study, metastable states pertaining to μ-opioid receptors (MORs) have been determined by means of Markov state model [85].

In above sections, GPCR monomers have been considered for the topics covered. However, it is also known that oligomerization has been widely used in GPCR family to carry out physiological functions. In the following section, we will review this phenomenon with an emphasis on GPCR-targeted drug development strategies.

### 3.2. Effect of Oligomerization on GPCR-Targeted Drug Discovery

**Figure 5:**
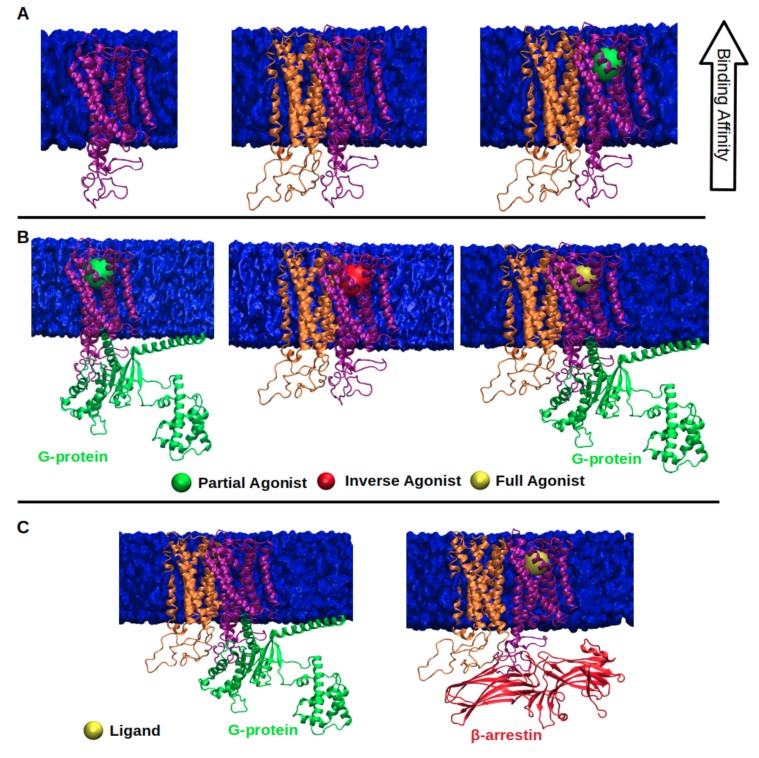
(**A**) depicts that ligand binding affinity of monomer (purple) increases in the presence of its partner protomer (orange); (**B**) ligand efficacy of monomer (purple) decreases (middle) or increases (right) in the presence of its partner (orange) when they form dimer; (**C**) functional selectivity of dimer changes in the presence of protomer; concurrently, G protein is substituted with β-arrestin.

The term “GPCR dimers or oligomers” was first coined by Fuxe et.al in 1991 [131]. Emergence of positive and negative cooperativity in GPCRs, which were delineated by experimental and computational studies, has further proved the existence of these oligomeric molecular assemblies. GPCR oligomerization leads to generation of novel signaling units with discrete pharmacological properties as a result of changes in properties of protomers, which is stemmed from the cross talk between individual receptors in the complex. Consequently, this allosteric communication either modulates binding affinity and/or efficacy of the ligand(s) that bind(s) to oligomer or introduces functional selectivity to one of the protomers as shown in Figure 5. For instance, when μ- and δ-opioid receptors are expressed together highly selective synthetic agonists for each of the protomer showed reduced potency and no sensitivity to pertussis toxin in contrast to individually expressed receptors, presumably due to interaction that is mediated with a different G protein subtype [132]. Similarly, affinity of D_2_R (Long form) for dopamine agonist and D_2_R signaling is dramatically reduced upon co-expression and stimulation of A_2A_R in membrane preparation of rat [133] and sheep striatum [31] due to antagonistic effect on D_2_R which is exerted by A_2A_R. This explains why A_2A_R antagonists increase both the affinity of dopamine for D_2_R and therapeutic efficacy of L-dopa, which is used in treatment of Parkinson’s disease. As to the functional selectivity, one of the protomers of the oligomer forces the other receptor unit to signal through a distinct signaling pathway. An example to this allosteric interaction can be given to D_2_R-ghrelin GHS1a heteromer where heteromerization of D_2_R with ghrelin GHS1a receptor in the hypothalamus modifies D_2_R signaling and leads to Gβγ-dependent mobilization of Ca^2+^ of the receptor [134]. Therefore, it has been thought that anorexigenic effect of D_2_R is caused by this heteromer, which might be used as a target for treatment of eating disorders.

From the above examples, it is indisputable that oligomer formation has dramatic effects on functional properties of individual GPCRs. Therefore, the oligomeric state of the receptor must be determined precisely, which can be done by using combination of experimental and computational tools [135], to target specific receptor assemblies. However, it is not a trivial task since interaction interfaces and/or partners within GPCR oligomers are changed under pathophysiological conditions or upon treatment with therapeutics as will be discussed below.

#### 3.2.1. Pathophysiological Conditions and Drug Therapy Modulate Interaction Interfaces/Partners in GPCR Oligomers

Lipid rafts are specific micro-domains of the plasma membrane which are rich in sphingolipid- and cholesterol. They actively participate in GPCR signaling and their roles in the pathogenesis of various diseases such as Alzheimer’s, Parkinson’s, liver fibrosis [136] and cardiovascular diseases have been revealed [137]. Considering that lipid rafts provide a suitable microenvironment for formation of GPCR signaling complexes, it is likely that identity of promoters in these assemblies changes under disease conditions. An example can be given to angiotensin AT_1_-cannabinoid CB_1_ heteromer. In normal liver angiotensin AT_1_ signals via Gq, whereas it forms heteromers with cannabinoid CB_1_ as a result of upregulation of the latter under alcohol-induced liver fibrosis. This heteromer formation causes a switch from G_q_- to G_i_-mediated signalling and can be blocked by CB_1_ receptor antagonist [32]. Another example is NTS1 heterodimerization with secretagogue receptor 1b, which is caused by aberrant overexpression of the receptor in cancerous cells. It causes a change in G protein selectivity of NTS1, which is implicated in lung cancer cell growth [138]. Apart from disease conditions, it has been shown that oligomerization is also used to control basal signaling level of NTS1, thus increasing the fidelity of GPCR signaling in the absence of agonist stimulus. Authors have shown that NTS1 participates in homo-oligomer formation when it is in the apo form by using multiple interaction interfaces, which are primarily mediated by TM5-6 and so not compatible with activation [139].

In addition to disease, drug therapy also affects the identity of protomers in the GPCR oligomer. Under physiological conditions, μ-opioid receptor forms homodimers and stimulation of the dimer with morphine induces G protein-mediated signaling with low β-arrestin-mediated signaling [140]. Upon chronic treatment with morphine, δ-opioid receptor expression is increased at the plasma membrane, and distribution of μ/δ-opioid heteromers is broadened [29,141]. Further morphine stimulation increases β-arrestin mediated signaling which brings about unwanted side effects such as tolerance to analgesic effects as well [142]. In another example, it has been shown that acute or chronic treatment of L-Dopa causes disruption of allosteric cross-talk in A_2A_R-Cannabinoid CB_1_-D_2_R heteromers in samples taken from striatum of hemiparkinsonian rats [34].

#### 3.2.2. Bivalent Ligands as Powerful Tools to Target GPCR Dimers

Bivalent ligands are made up of two pharmacophores, each of which is specific to the protomer that makes up the GPCR dimer, and are connected with an appropriate linker group. They provide simultaneous occupation of both receptors, thus selective targeting of the GPCR pair. On top of selectivity, they also enhance efficacy of protomers rather than individual administration of both ligands. It has been shown that bivalent ligands which are composed of opioid alkaloids and peptide agonists derived from enkephalins have been shown to possess increased opioid potency and selectivity as compared to corresponding individual counterparts [143]. In another example, it has been shown that dimerization of κ-opioid receptor with NTSR1 results with a switch from G protein to β-arrestin2 signalling that is mediated by κ-opioid receptor. In addition, it has been found out that dual occupancy of the dimer puts β-arrestin2-mediated signaling back to G_i_ protein-dependent one. This finding suggests that development of a bivalent ligand, which is composed of κ-opioid receptor antagonist and NTSR1 agonist as protomers, should enhance analgesic property of κ-opioid receptor, thus relieving existing side effects [144].

## 4. Selective Targeting of Effector Subtype Is Crucial for Development of Safer Treatment Strategies

GPCRs couple to four subtypes of G proteins (Gαs, Gαi/o, Gαq/11, Gα12/13) [145] and four subtypes of Arrestins (Arrestin1, 2, 3 and 4) [146]. In a recent paper, it has been shown that Gαi and Gαs display different modes of G protein binding [42]. From a structural perspective, this relates to exposure of different regions of the effector to the cytosol. Consequently, receptor-effector complex can bind to different downstream partners and initiate distinct signaling pathways. Therefore, it is obvious that specific targeting of G protein or Arrestin subtypes is crucial for developing safer treatment strategies. Here, we focus on Arrestin but similar examples can also be given for G protein as well. Arrestin protein family is small such that it is composed of only four members. In addition, they display remarkable differences in their functions. Specifically, Arrestin1 and 4, which are also known as visual arrestins, exclusively bind to activated and phosphorylated Rhodopsin and terminate light-activated phototransduction in rod and cone cells. However, Arrestin2 (β-arrestin1) and Arrestin3 (β-arrestin2) are ubiquitous, outside the retina, and participate in many physiological processes by interacting with various types of GPCRs. In that sense, Arrestin3 is even more interesting as it can bind active and phosphorylated/non-phosphorylated GPCRs depending on the type of the receptor [147] in spite of sharing a high sequence similarity and conserved structural fold within the Arrestin family. A possible activation mechanism has been proposed that can explain this functional difference and can be exploited to target specific Arrestin subtypes [148].

The importance of selective targeting of Arrestin subtypes can be understood when considering certain types of cancer. Specifically, Arrestin2 participates in cell propagation and senescence in acute lymphoblastic leukemia [149], cell proliferation in gastric cancer [150]. Obestatin stimulates Akt signalling in gastric cancer cells through β-arrestin-mediated epidermal growth factor receptor transactivation), and cell migration in melanoma [151], whereas Arrestin3 does not contribute to these processes. However, Arrestin3 triggers cell growth and metastasis in renal cancer [152] as well as cell proliferation and invasion in pancreatic cancer [153]. Another example can be given for heart diseases, Arrestin2 promotes negative inotropy as well as adverse remodeling post-myocardial infarction [154]. On the other hand, Arrestin3 has anti-apoptotic and anti-inflammatory effects which attenuates post-myocardial adverse remodeling as opposed to Arrestin2. Lastly, asthma can be given as another example, where arrestin subtype selectivity is crucial. It has been shown that Arrestin3 causes development of allergic inflammation [155] at an early step in the inflammatory cascade, so novel therapeutic molecules that target this protein may help treatment of asthma. To sum up, activation/inhibition of relevant Arrestin subtypes can be achieved by utilizing differences in activation mechanism of Arrestin subtypes as mentioned above, thus building a platform for development of safer drugs with reduced side effects.

## 5. Understanding Impact of Single Nucleotide Polymorphism Improves Personalized Medicine Strategies

**Figure 6:**
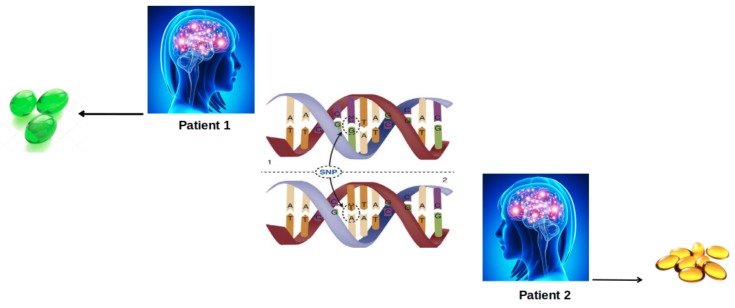
The effect of SNP on drug response. A single nucleotide variation causes two patients with the same disease to be prescribed differently as indicated by different colors and numbers of pills.

In spite of being one of the most studied drug targets, the impact of pharmacogenetics pertaining GPCRs, GPCR kinases, G proteins and Arrestins has remained elusive. That is to say, knowledge regarding genetic background of these proteins has not been included yet as routine in drug discovery studies. On the other hand, studies have shown that single nucleotide polymorphism (SNP) impacts the drug response (see Figure 6). Depending on the location, these nucleotide variations might influence activation, allostery and function of the protein, thus affecting efficacy of the drug and also susceptibility of the patient to adverse reactions. In particular, these sites might be ligand binding sites, allosteric sodium binding sites, micro-switches, and cytosolic effector coupling sites. However, additional polymorphisms have been also observed in regions with unknown functional impact [21] and it is more difficult to predict the phenotypic outcome of a nucleotide variation if it is located in a region with unknown function.

Dopamine 3 receptor is a good representative system where receptor associated SNPs impact the drug response. Specifically, the SNP seen in Dopamine 3 receptor has been shown to linked to increased risk of gastrointestinal toxicity in patients having Parkinson’s disease upon L-Dopa treatment [22]. Besides GPCRs, a nucleotide variation at a specific Arrestin3 gene locus causes some patients having Parkinson’s disease to give lower response to antidepressants than the others [23].

From above examples, it is indisputable that SNPs cause unpredictable side effects. To circumvent this problem, studies which are centered on characterization of these variants must be expedited. In this direction, an online and interactive platform, which allows researchers to investigate the impact of these variations on a given drug target, has been incorporated into the GPCR database [156]. In addition, if the SNP causes an amino acid change in the protein, wild type and variant forms of the target can be subjected to molecular dynamics simulations to investigate impact of this amino acid change on structure and dynamics of the protein. Consequently, key regions which are responsible for variant-specific dynamics can be determined and then targeted, for instance, by means of therapeutic molecules to mimic properties of wild type proteins.

## 6. Concluding Remarks and Perspectives

Involvement in almost every physiological process in the cell has made GPCRs one of the most studied drug targets in the drug market. The two breakthroughs in the GPCR field, in particular, β-arrestin mediated but G protein independent signaling and also separability of G protein- and β-arrestin-mediated signaling pathways have shifted the research focus into development of novel strategies that are used for specific targeting of these signaling pathways. Here, the motivation is to alleviate possible side effects which are resulted from simultaneous targeting of both G protein- and β-arrestin-mediated signaling pathways. In this review, we summarize the current state of biased GPCR signaling with a focus on their therapeutic potential in diseases. We discuss several methods which can be used to enrich a certain downstream signaling pathway among many others. However, this is not trivial as the outcome strongly depends on the genetic background of patients. That is to say, single nucleotide polymorphisms seen in GPCRs and/or GPCR-related proteins impact the response elicited by GPCR-targeted drugs. Therefore, this necessitates inclusion of pharmacogenomics in GPCR-targeted drug discovery studies. To this end, all possible polymorphic sequences can be predicted and investigated a priori to check if they overlap with regions with functional relevance. In any case, these variants can be modeled and investigated at an atomistic level by means of molecular dynamics simulations to have a holistic understanding [135] of mechanistic impact of these polymorphisms on both structure and dynamics of proteins, which expedite systematic and precise targeting of GPCRs and related proteins and building a platform for development of successful personalized medicines. Consequently, quality of life of patients is improved while economic burden is attenuated.
